# In-Situ Thermoresponsive Hydrogel Containing Resveratrol-Loaded Nanoparticles as a Localized Drug Delivery Platform for Dry Eye Disease

**DOI:** 10.3390/antiox12050993

**Published:** 2023-04-25

**Authors:** Ilenia De Luca, Francesca Di Cristo, Raffaele Conte, Gianfranco Peluso, Pierfrancesco Cerruti, Anna Calarco

**Affiliations:** 1 Research Institute on Terrestrial Ecosystems (IRET)—CNR, Via Pietro Castellino 111, 80131 Napoli, Italy; ilenia.deluca@iret.cnr.it (I.D.L.);; 2 Elleva Pharma s.r.l., Via P. Castellino 111, 80131 Napoli, Italy; francesca.dicristo2@gmail.com (F.D.C.); raffaele.conte86@tiscali.it (R.C.); 3 Faculty of Medicine and Surgery, UniCamillus-Saint Camillus International University of Health Sciences, Via di Sant’Alessandro 8, 00131 Rome, Italy; 4 Institute for Polymers, Composites and Biomaterials (IPCB-CNR), Via Campi Flegrei 34, 80078 Pozzuoli, Italy

**Keywords:** eye dry disease, resveratrol, acetylated nanoparticles, hydrogel, epithelial corneal cells, antioxidant, anti-inflammatory

## Abstract

Dry eye disease (DED) is a dynamic and complex disease that can cause significant damage to the ocular surface and discomfort, compromising the patient’s quality of life. Phytochemicals such as resveratrol have received increasing attention due to their ability to interfere with multiple pathways related to these diseases. However, the low bioavailability and the poor therapeutic response of resveratrol hinder its clinical applications. Cationic polymeric nanoparticles, in combination with in situ gelling polymers, could represent a promising strategy to prolong drug corneal residence time reducing the frequency of administration and increasing the therapeutic response. Eyedrop formulations, based on acetylated polyethyleneimine-modified polylactic-co-glicolyc acid- (PLGA-PEI) nanoparticles loaded with resveratrol (RSV-NPs) were dispersed into poloxamer 407 hydrogel and characterized in terms of pH, gelation time, rheological properties, in vitro drugs release, and biocompatibility. Moreover, the antioxidant and anti-inflammatory effects of RSV were assessed in vitro by mimicking a DED condition through the exposition of epithelial corneal cells to a hyperosmotic state. This formulation exhibited sustained release of RSV for up to 3 days, exerting potent antioxidant and anti-inflammatory effects on corneal epithelial cells. In addition, RSV reversed the mitochondrial dysfunction mediated by high osmotic pressure, leading to upregulated sirtuin-1 (SIRT1) expression, an essential regulator of mitochondrial function. These results suggest the potential of eyedrop formulation as a platform to overcome the rapid clearance of current solutions for treating various inflammation- and oxidative stress-related diseases such as DED.

## 1. Introduction

Eyes are constantly exposed to environmental factors (e.g., air pollutants or adverse indoor and/or outdoor environmental conditions, light exposure, ultraviolet rays, ionizing radiation) that compromise tear film composition and ocular surface components [1]. Prolonged exposure to these insults triggers oxidative damage of the ocular surface through the production of reactive oxygen species (ROS) and activation of pro-inflammatory signaling pathways [2,3]. Overproduction of ROS in tear and ocular surface structures represents one of the main characteristics of Dry Eye Disease (DED) [3], a multifactorial disease quite frequent in the elderly population, and growing rapidly in young people as well [4]. The loss of tear film homeostasis and the damage of the affected eye area up to the cornea causes visual discomfort and disturbance to the patient often compromising daily life [5]. DED symptoms include burning, inflammation, foreign body sensation, blurred vision, heaviness and tear film instability. According to the multifactorial and wide spectrum of DED etiology, the treatment must be tailored specifically to each patient by targeting the specific mechanisms in accordance with disease severity. Mild DED cases are treated with artificial tears to reduce tear evaporation and corneal surface inflammation. More serious cases require treatment with anti-inflammatory agents, such as topical corticosteroids, systemic cholinergic agents, and autologous serum tears [6]. However, long-lasting application of ocular preparations containing preservatives (e.g., benzalkonium chloride (BAK), chlorobutanol, sodium perborate), may cause side effects due to the disruption of the ocular epithelial cell-cell junction and toxic epitheliopathy in the case of preserved products Hence, there is still a demand for novel therapeutic approaches.

Plant polyphenols are vegetable secondary metabolites able to promote beneficial health effects, such as anti-inflammatory, antioxidant and antimicrobial [7]. In particular, their bioactivity is able to influence both the production of ROS, IL (interleukins), ELAM-1 (endothelial leucocyte adhesion molecule-1), the expression of the TNF-α (tumor necrosis factor-α), and VEGF (vascular endothelial growth factor) pathways in ocular tissues. Moreover, some polyphenols could increase antioxidant defense systems and inhibit p53-dependent apoptosis involved in age-related eye disease [8,9]. Moreover, it has been reported that oxidative stress may directly affect ocular surface health, inducing DED. Therefore, topical/systemic use of antioxidants represents a promising approach to the treatment of DED [10].

Recent in vivo and in vitro studies demonstrated that curcumin, epigallocatechin gallate, resveratrol, quercetin, betaine and pterostilbene polyphenols are effective in DED pathology treatment [11]. Among all polyphenols, increasing attention is being paid to Resveratrol (RSV, 3,5,4′-trihydroxy-trans-stilbene) due to its strong antioxidant and anti-inflammatory effects also in ophthalmologic diseases [12,13]. Further, RSV regulates inflammatory responses via various signaling pathways in a dose-dependent manner. Moreover, RSV acts as an antioxidant by controlling antioxidant enzymes and blocking free radical damage to DNA [14].

Even though several studies demonstrated RSV’s potential applicability as a treatment for DED, its low water solubility, low stability and poor ocular bioavailability limit clinical efficacy. Recent studies focused on nanotechnology approaches to overcame these limits and produce ophthalmic polyphenols-enriched formulations [15]. Compared to currently available approaches for administering eye drops, nanostructured polymers with bioadhesive properties (e.g., cationic nanoparticles) represent a more efficient approach to delivering the appropriate concentrations of bioactive molecules to the eye. Positively charged nanocarriers could interact with negatively charged cells of the ocular surface prolonging the residence time of the administered eye drops [16]. Hence, the present study aims to design an in situ thermogelling formulation containing RSV-loaded polymeric nanoparticles able to ameliorate the clearance of current eye drop solutions. To this purpose, cationic acetylated lactide/glycolide-polyethyleneimine (PLGA-PEI) nanoparticles (AcPEI-NPs) loaded with RSV were prepared by nanoprecipitation and incorporated in the Poloxamer 407 thermoresponsive hydrogel (RSV@Tgel) to develop novel devices for the treatment of ocular diseases. Poloxamer 407 is a member of the Pluronics^®^ triblock copolymers, made of polyethylene glycol-*b*-polypropylene glycol-*b*-polyethylene glycol, which are non-ionic, water-soluble materials widely used as pharmaceutical excipients [17]. It has been demonstrated that the use of polymers that exhibit thermoresponsive sol-to-gel phase transition in the cul-de-sac could overcome the loss of drug in precorneal tissue caused by eye drainage, thereby extending drug-cornea contact time. Indeed, these ophthalmic formulations, by changing their structure and viscosity upon heating, can be administered as a liquid, and then form a gel in specific environmental circumstances, such as in contact with the eye [18]. The RSV@Tgel formulation was physiochemically characterized, and the release profiles of RSV were evaluated in simulated tear fluid. Moreover, the role of released RSV in preventing oxidative stress and cytokine release was explored in human corneal epithelial cells (HCECs) under hyperosmotic stress, as an in vitro dry eye model. The reported results demonstrated the ability of RSV@Tgel to attenuate hyperosmolar-induced mitochondrial dysfunction in HCECs, restoring mitochondrial function by inducing SIRT1 expression. For these reasons, RSV@Tgel could be an excellent adjuvant in dry eye disease due to its protective effect against inflammation and oxidative stress, as well as the reduced number of eye drop instillations.

## 2. Materials and Methods

### 2.1. Materials

d,l-Lactide/glycolide copolymer (PLGA, PURASORB^®^, inherent viscosity 0.20 dL/g) was a generous gift from PURAC (Gorinchem, The Netherlands). Branched polyethylenimine (PEI, MW: 25 kDa), acetic anhydride, dicyclohexylcarbodiimide (DCC), N-hydroxysuccinimide (NHS), acetone, resveratrol (RSV), polyvinyl alcohol (PVA, average mol wt. 30,000–70,000), Poloxamer 407, phenylmethylsulfonyl fluoride (PMSF), benzamidine, and hydroquinone (HQ) were obtained from Sigma-Aldrich (Milan, Italy). All HPLC analytical grade solvents were purchased from Fisher Scientific (Milan, Italy). Dulbecco’s modified Eagle medium (DMEM; Euroclone, Milan, Italy) containing 10% fetal bovine serum (FBS; Euroclone), 100 U/mL penicillin, and 100 µg/mL streptomycin (Euroclone), in 5% CO_2_ at 37 °C and 95% humidified room air.

### 2.2. Physico-Chemical Characterization of RSV-Loaded Nanoparticles (RSV-NPs)

Acetylated nanoparticles (AcPEI-PLGA) were prepared using a two-step procedure as reported by Conte et al. with modifications [19]. Briefly, AcPEI-PLGA copolymer (50 mg) and different quantities of RSV (1–10 mg) were completely dissolved in 10 mL of acetone under ultrasound to form a homogeneous mixed system (Digital Sonifier S-250D, BRANSON, Milan, Italy). The resultant solution was added dropwise into the aqueous phase, containing 100 mL PVA (0.5% *w*/*v*), and stirred overnight on a magnetic stir plate (at 300 rpm) to allow evaporation of the organic solvent. The RSV-NPs were collected by centrifugation for 45 min at 14,810× *g*, 4 °C (5718R, OHAUS, Milan, Italy), washed with ultrapure water (Milli-Q^®^ IQ 7000, Merk Life Science S.r.l., Milan, Italy) and lyophilized. Control nanoparticles (NPs) were prepared following a similar procedure omitting RSV. The hydrodynamic diameter, zeta potential, and polydispersity index (PDI) were measured using Dynamic Light Scattering (DLS, Malvern Zetasizer, Malvern Instruments Ltd., Malvern, UK), and the particle concentration was assessed by Nanoparticles Tracking Analysis (NTA-NanoSight NS300, Malvern Instruments Ltd., Malvern, UK) as reported by Valentino et al. [20]. For both analyses, experiments were performed at 25 °C using a sample volume of 1 mL. The average values were obtained with the data from six separate measurements.

The amount of drug entrapped in NPs was determined indirectly, in triplicate, by analyzing the amount of free RSV in the supernatant (18,620× *g* for 30 min; 5718R, OHAUS, Milan, Italy) as described in Section 2.6. The encapsulation efficiency of RSV-NPs was determined based on the following equation:

(1) $$\mathrm{EE}\%\;=\frac{\mathrm{Total}\;\mathrm{amount}\;\mathrm{of}\;\mathrm{drug}\;-\;\mathrm{Amount}\;\mathrm{of}\;\mathrm{drug}\;\mathrm{in}\;\mathrm{supernatant}}{\mathrm{Total}\;\mathrm{amount}\;\mathrm{of}\;\mathrm{drug}}\times 100$$

### 2.3. Mucoadhesion of RSV-NPs

The interaction between mucin and RSV-NPs was evaluated by mixing equal volumes of mucin (0.5 mg/mL in PBS) and NPs (2.53 × 10^9^ particles/mL) at 35 °C for 2 h [21]. The suspension was, then, centrifuged at 15,600× *g* (Corning LSE high speed microcentrifuge; Fisher Scientific Italia, Milan, Italy) for 1h at room temperature (25 °C). UV-Visible spectrophotometer (UV-1650 PC, Shimadzu, Milan, Italy) at 255 nm was used to quantify the amount of free mucin. All nanoformulations were analyzed in triplicate (*n* = 3). The mucin-binding efficiency of NPs (%) or mucoadhesive strength of NPs was calculated by using the equation stated as follows:

(2) $$\mathrm{Mucoadhesive}\;\mathrm{efficiency}\;\left(\%\right)=\frac{\mathrm{Total}\;\mathrm{amoun}\;\mathrm{of}\;\mathrm{mucin}-\mathrm{Free}\;\mathrm{amount}\;\mathrm{of}\;\mathrm{mucin}}{\mathrm{Total}\;\mathrm{amount}\;\mathrm{of}\;\mathrm{mucin}}\times 100$$

### 2.4. Preparation of RSV-Loaded Hydrogels (RSV@Tgels)

Poloxamer gels were prepared by the cold method as described by Matthew et al. [22] with slight modifications. Briefly, an appropriate amount of polymer was accurately weighed and dispersed in purified distilled water with continuous mild stirring (until polymer dissolution) to prepare solutions at varying polymer concentrations (15–22% *w*/*v*). The poloxamer 407 concentration was chosen based on gelation temperature and gelation time. To obtain RSV-NPs dispersion in gel (RSV@Tgel), weighed amounts of lyophilized RSV-NPs (0.1, 0.5, and 1 mg/10 mL solution) were homogeneously mixed with cold poloxamer 407 overnight in an ice bath.

### 2.5. RSV@Tgels Characterization

#### 2.5.1. Rheological and Mechanical Studies

The sol-gel transition of the RSV@Tgels containing 1 mg/10 mL RSV-NPs was determined by non-isothermal oscillatory rotational rheometry, by means of a Thermo Scientific Mars III device equipped with steel parallel plates (20 mm, plate gap = 1.0 mm). The measurements were carried out at an angular frequency of 10 rad/s and varying deformation from 0.5 to 5%, with a heating rate of 1 °C/min from 20 to 50 °C. Storage (G′) and viscous moduli (G″) were recorded as a function of temperature. The temperature at which G′ attained the plateau value was considered as the gelling temperature, T_gel_. At least three replicates were performed. Compressive stress–strain tests were performed using the same equipment, with a loading-unloading strain rate of 0.6 mm/min, and a strain level of up to ≈20% of the original plate gap. The compressive moduli were obtained by linear fitting of the stress–strain curves in a strain range of 2–5%. In the cyclic loading–unloading tests, compression and load were recorded for 10 cycles. Each reported value was the mean of at least three measurements.

#### 2.5.2. Gelation Time

The gelation time (t_sol-gel_) of RSV@Tgels was determined by a modified test tube inversion method using simulated tear fluid (0.67 g NaCl, 0.2 g NaHCO_3_, and 0.008 CaCl_2_·2H_2_O in 100 g of purified water, STF) as gelation solution [23]. Briefly, a glass vial containing 1.0 mL of sample was kept at the storage temperature of 4 °C for 2 h, then withdrawn and placed in a water bath set at 34 °C. The glass vial was then repeatedly turned upside down to check for gelling of the sample. The gelation time represents the temperature at which the gel did not exhibit gravitational flow during a period of 2 min when the tube was reversed.

#### 2.5.3. Short-Term Stability Studies

The physicochemical stability of RSV@Tgels was monitored over a period of 14 days of storage at different temperatures (4.0 ± 0.5 °C and 25.0 ± 0.5 °C) by visual observation and pH determination. At predetermined times (0 and 14 days), nanoparticles were separated from the hydrogel by centrifugation (12,000× *g*, 2 h; Corning LSE high speed microcentrifuge; Fisher Scientific Italia, Milan, Italy). The physical stability of the formulation was visually examined for its dispersion parameters such as the presence of aggregates and the occurrence of coalescence phenomena. Further, all samples were analyzed for particle size, PDI, and % drug entrapment efficiency, and the results were compared with the initial values. The RSV stability during storage was confirmed by HPLC analysis as described in Section 2.6.

### 2.6. In Vitro RSV Release and Permeation Studies

The cumulative RSV release was assessed in 5 mL of freshly prepared STF at two different osmolarity values, namely 312 and 500 mOsm/L. During the release test, the temperature was maintained at 34.0 ± 0.5 °C under continuous magnetic stirring (200 rpm). At given time intervals, 0.5 mL of release media was withdrawn and replenished with fresh media. Samples were centrifuged for 45 min at 21,380× *g* (Corning LSE high speed microcentrifuge; Fisher Scientific Italia, Milan, Italy) and the amount of RSV in the samples was determined by liquid chromatography–tandem mass spectrometry (LC-MS/MS), as indicated by Amaghnouje et al. [24].

The permeation studies were performed using vertical Franz diffusion cells with a volume of 12 mL (contact area: 1.77 cm^2^, PermeGear Inc., Hellertown, PA, USA) as reported by Bao et al. [25]. Cellulose acetate membranes were placed on top of the receptor chambers of the cells following the addition of freshly prepared STF as release media. During the release test, the temperature was maintained at 34.0 ± 0.5 °C. To simulate the small amount of tear secreted on the eye surface, 0.25 mL of the release medium was added to the top of the samples loaded into the donor chambers. At specified time intervals, aliquots (0.15 mL) of fresh media were used to replace the same volume withdrawn from the receptor chambers. Then, the supernatants were directly assayed by LC-MS/MS after centrifugation at 21,380× *g* (Corning LSE high speed microcentrifuge; Fisher Scientific Italia, Milan, Italy), 4 °C for 45 min.

### 2.7. In Vitro Cell Studies

#### 2.7.1. Cells Culture and In Vitro Dry Eye Model

Human Corneal Epithelial Cells (HCECs) were obtained from the American Type Culture Collection (CRL-11135, ATCC, Milan, Italy) and maintained in DMEM supplemented with 10% FBS, 4.5 mg/mL glucose, 2.0 mM glutamine, 100 U/mL penicillin, 100 µg/mL streptomycin, and 10 ng/mL human epidermal growth factor in a humidified atmosphere of 5% CO_2_–95% air and 37 °C. HCECs from the third to tenth passages that exhibited good morphology were used for experiments. The cell viability of HCECs on RSV@Tgel was assessed following the international standard ISO 10993 by cell counting kit (CCK-8) according to the manufacturer’s protocol (Sigma-Aldrich, Milan, Italy). Briefly, the gel samples were immersed and incubated in DMEM at 37 °C for 24 h to obtain the “conditioned medium” (CM). Following the adherence of HCECs, the culture medium was removed and replaced with 200 μL CM for 6, 12, and 24 h. Then, 10 μL of CCK-8 solution was added to each well and the absorbance was measured at 450 nm. Cell viability was determined as a percentage compared to the untreated cells.

Cellular death by membrane damage was measured using Lactate Dehydrogenase (LDH) assay (Sigma-Aldrich) as per the manufacturer’s instructions. Cells were treated as previously described and LDH released in the culture medium was measured at 490 nm. Triton X-100 solutions (1%) and culture media only were used as positive and negative controls, respectively.

For Dry Eye Cell Model, HCECs were treated with culture medium at different osmolarities ranging from 312 to 500 mOsm/L as reported by Shetty et al. [26]. The protective effects of RSV were studied with the acute toxicity model by pre-treating cells with RSV@Tgel for 24 h. A shorter exposure (4 h) was used to investigate the effects on mRNA expression.

#### 2.7.2. Intracellular Antioxidant Activities

As previously reported [27], the CM-H_2_DCFDA assay kit (Sigma-Aldrich) was used to determine intracellular ROS production according to the manufacturer’s protocol.

To assess antioxidant enzyme activities, HCECs were treated as reported above and resuspended in 0.5 mL PBS buffer containing 0.5 mM phenylmethylsulfonyl fluoride (PMSF) and 0.2 mM benzamidine. Measurements of Catalase (CAT) and Superoxide dismutases (SODs) activities were carried out on cell lysates after sonication (three intervals of 5 s). Hydrogen peroxide (H_2_O_2_) decomposition (at 240 nm) was followed to determine CAT activity (EC 1.11.1.6). Results were normalized to total protein content and expressed as a percentage of control (100% of activity). SOD (EC 1.15.1.1) activity was measured using the standard assay mixture containing an enzymatic sample as per the manufacturer’s instructions (Sigma-Aldrich). Samples were exposed for 5 min to intense cool white light. One SOD unit was defined as the amount of enzyme necessary to inhibit 50% of the reaction rate. Samples were measured at 560 nm in a Cytation 3 microplate reader. Results were normalized to total protein content and expressed as a percentage of control. Experiments were performed four times.

#### 2.7.3. Enzyme-Linked Immunosorbent Assay (ELISA)

The levels of human interleukin-6 (IL-6), human interleukin-8 (IL-8), and tumor necrosis factor (TNF-*α*) were measured in supernatants using an Enzyme-Linked Immunosorbent Assay (ELISA) as reported by Conte et al. [28].

#### 2.7.4. RNA Isolation, Reverse Transcription, and Quantitative Real-Time PCR (qRT-PCR)

Total RNA was extracted from cell cultures using TriFast (EuroClone, Milan, Italy), according to the manufacturer’s protocol, and mRNA levels were measured by RT-PCR amplification as reported by De Luca et al. [29]. Specific primers for Human Matrix Metallopeptidase 9 (*MMP-9*), Interleukin-6 (*IL-6*), Interleukin-8 (*IL-8*), tumor necrosis factor (*TNF)-α*, Sirtuin 1 *(Sirt1)* and β-Actin (*ACTB*) were used and listed in Table 1. All reactions were run in triplicate, normalized to the housekeeping gene (*ACTB*), and the results were expressed as mean ± SD. The 2^−ΔΔCt^ method was used to determine the relative quantification.

### 2.8. Measurement of Cellular Respiration

Analysis of mitochondrial function was performed on live cells using a Seahorse Biosciences XFe24 Flux Analyzer (Seahorse Biosciences, Milan, Italy) according to the manufacturer’s protocol. HCECs (2 × 10^4^/well) were seeded in an XF 24-well plate and grown until confluence. Then, cells were treated with a hyperosmotic medium with or without RSV@Tgels CM (100 µL) for 2 h. The cells were washed twice with XF base medium supplemented and incubated in a CO_2_-free environment for 60 min at 37 °C and then placed in the XFe24 Flux Analyzer [30]. Oxygen consumption rate (OCR) was measured under basal conditions followed by the sequential addition of 1 µM oligomycin to inhibit adenosine triphosphatase (ATP) synthase activity, 1 µM tri-fluorocarbonylcyanide phenylhydrazone (FCCP) to uncouple mitochondrial oxidative phosphorylation, and 1 µM antimycin A and rotenone to inhibit electron transport in complex I and III, respectively. ATP turnover rate was the difference in OCR between the last measurement before the oligomycin injection and the minimum OCR after the oligomycin injection. Maximal respiration was the difference in OCR between FCCP-induced respiration and OCR after the injection of antimycin A. Spare respiratory capacity was the difference between maximal respiration and basal OCR. OCRs were normalized to total protein content quantified with a BCA protein assay.

### 2.9. Western Blotting

Total proteins of HCECs were extracted in ice-cold RIPA buffer (1:10 *w*/*v*) containing protease inhibitor. Then, Western blot analysis was carried out as described in our previous studies [31,32] using a mouse monoclonal Sirt1 antibody (sc-74465, 1:500, Santa Cruz Biotechnology, Milan, Italy), followed by a secondary antibody conjugated with horseradish peroxidase. The bands were quantified using ImageJ analyzing software. β-actin (ACTB) was used as an internal reference.

### 2.10. Statistical Analysis

Data were presented in means ± SD of at least three independent experiments. Statistical comparisons between the different experimental groups and their corresponding controls were made with a one-way analysis of variance (ANOVA) followed by Tukey’s post hoc test and Student’s *t*-test, considering *p* < 0.05 as statistically significant. All the data were analyzed with the GraphPad Prism version 8.01 statistical software package (GraphPad Software, San Diego, CA, USA).

## 3. Results and Discussion

### 3.1. Preparation and Characterization of RSV-NPs

Eye-drops and gels represent the most common and convenient topical ophthalmic formulations for ocular delivery, owing to the simple fabrication and the easy/noninvasive administration route. Despite their popularity among patients, drugs delivered through this route are usually poorly absorbed because of the short residence time in the mucosa and corneal permeability [33]. Nanocarrier-based therapeutic delivery systems have been developed to increase the corneal retention of drugs with the final goal of improving the efficacy of treatments for different ocular diseases.

**Figure 1:**
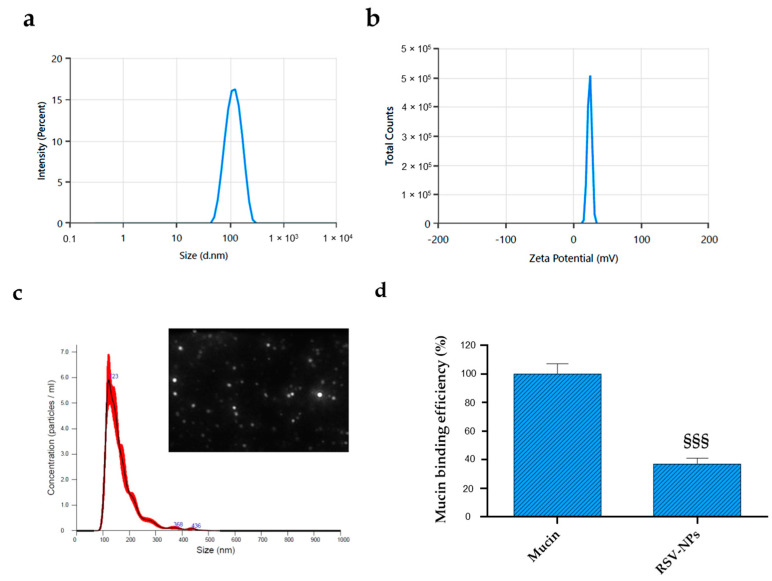
Physico-chemical properties of RSV-NPs. (**a**) Intensity-based size distribution, and (**b**) zeta potential profile of RSV-NPs. (**c**) NTA measurements for RSV-NPs in suspension and screenshot of representative NTA video. Hydrodynamic diameter distribution curves and zeta potential are average of three measurements. (**d**) Mucin binding efficiency (*n* = 3 ± S.D.—Student’s *t*-test. §§§ *p* < 0.001 shows significant difference with respect to mucin alone.

To accomplish prolonged contact time and hence increased residence time in the eye, in this study, PLGA was functionalized with PEI, a biocompatible, biodegradable cationic polymer. Several studies report that positively charged nanoparticles can enhance drug residence time by interacting with anionic tear-film mucins and with the negatively charged surface of cornea and conjunctiva, improving drug bioavailability [16,34,35]. The tertiary amines of PEI were partially acetylated (AcPEI) to improve PEI tolerability while maintaining a positive zeta potential. Indeed, particle size and surface charge are the most important parameters in ophthalmic formulations to warrant low irritation, sufficient bioavailability, and good compatibility with ocular tissues. AcPEI-PLGA nanoparticles loaded with RSV (RSV-NPs) were prepared by a modified nanoprecipitation method, using acetone as a suitable solvent. Nanoprecipitation represents a simple and reproducible technique that possesses a limited number of controllable variables and can be easily scaled up [35]. The prepared NPs were characterized by DLS, zeta potential measurements, and NTA (Figure 1a–c). As reported in Table 2, the lower drug/polymer ratio led to small nanoparticle sizes (89.2 ± 0.9), whereas the higher drug/polymer ratio tended to increase nanoparticle sizes (148.7 ± 2.2) and reduce the EE% (66.4 ± 5.9). This indicated that there was an optimal drug/polymer ratio to reach the maximum EE%, set at 1:10. Moreover, the different drug/polymer ratios have no significant effect on the polydispersity index and zeta potential. Furthermore, the polydispersity of all batches was below 0.3, indicating uniform particle size distribution, while the zeta potential greater than 20 mV suggested a high colloidal stability of RSV-NPs due to the electrostatic repulsion. Indeed, the higher the absolute value of zeta potential, the more stable the system [36].

Although the size obtained for all formulations agreed with ocular drug release requirements (≤200 nm), RSV_2_-NP (coded RSV-NPs), was the formulation selected for further characterization because of the higher EE (Figure 1). The RSV-NPs intensity-based size curve showed a monomodal and narrow distribution (Figure 1a), confirmed by NTA results which also provided the NPs concentration (Figure 1c). In particular, more than 50% of NPs were in a size range between 100 and 150 nm, and the concentration of the NPs with a 100 to 123 nm hydrodynamic diameter was about 2 × 10^8^ particles/mL.

The blink reflex and tear turnover rate are the main contributing factors to a short ocular retention time [37]. The use of cationic nanoparticles represents a possible strategy to extend the residence time and improve the bioavailability of the encapsulated molecule on the ocular surface. Whereas the mucin-aqueous layer of the tear film is commonly reduced in DED patients, leading to inflammation, and patient discomfort [38,39], the cationic nature of RSV-NPs could prolong the residence time in the ocular region and consequently improve RSV bioavailability and therapeutic action. As shown in Figure 1d, cationic RSV-NPs are able to interact with negatively charged sialic acid residues in the mucus with a mucin-binding efficiency of 63% at the ocular pH.

### 3.2. Rheological and Mechanical Characterization of Hydrogel Formulations (RSV@Tgel)

Liquid formulations based on polymers able to undergo a thermally induced sol-to-gel phase transition can overcome the issue related to removal from the eye through blinking, tear production, and nasolacrimal drainage. In the case of ophthalmic formulation, an optimal gelation temperature should be in the 32–34 °C range. Formation of the hydrogel at temperatures lower than 30 °C limits manufacturing, and handling, and causes difficulty in administration. In contrast, a gelation temperature higher than 36 °C entails that the formulation maintains its liquid state at body temperature, resulting in rapid elimination after administration [40]. Additionally, values of storage modulus G′ above 1000 Pa provide evidence of a pronounced elastic, solid-like character, which is highly sought in formulations employed for ocular administration to avoid washout from the corneal surface [41].

**Figure 2:**
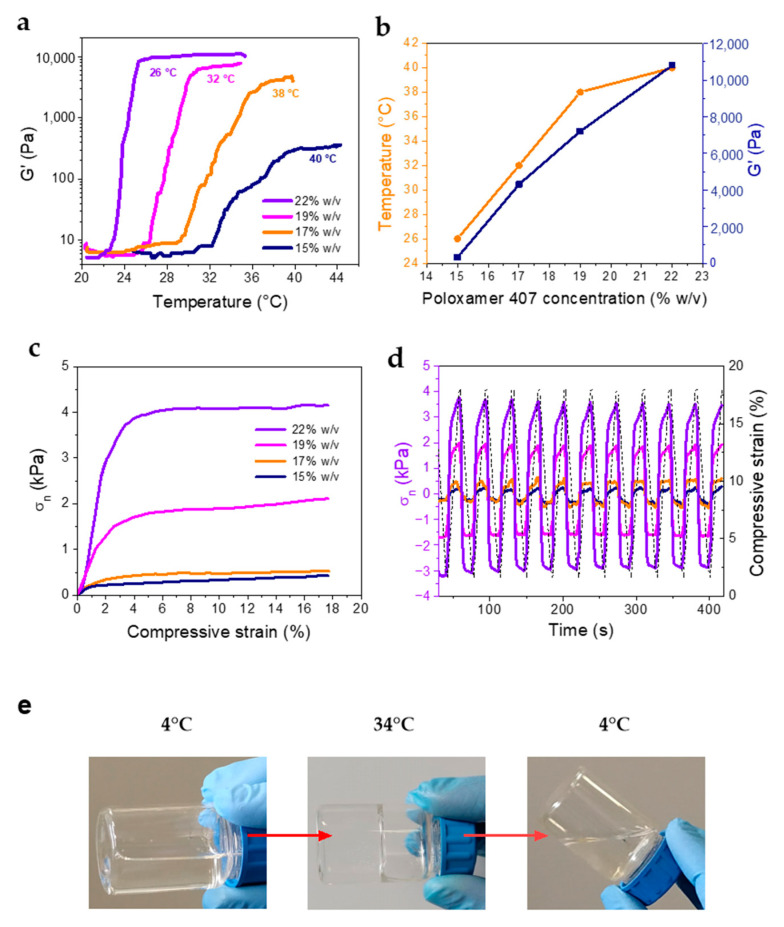
Rheological and mechanical characterization of the RSV@Tgel formulations containing 15, 17, 19, or 22% *w*/*v* poloxamer 407. (**a**) Evolution of the dynamic storage modulus G′ in non-isothermal oscillatory shear experiments. (**b**) T_gel_ and related G′ values as a function of poloxamer 407 concentration. (**c**) Representative compressive stress/strain curves, and (**d**) cyclic loading-unloading curves of the RSV@Tgel formulations. (**e**) Visual observation of the reversible gelling behavior of RSV@Tgel (19%) at 4 and 34 °C.

Therefore, the evaluation of the gelling temperature (T_gel_) is a fundamental prerequisite for the development of an effective ophthalmic formulation. In this respect, several formulations of RSV-NPs in the form of in-situ gelling solutions were prepared by varying the concentration of poloxamer 407 (15–22% *w*/*v*) aiming at protracting drug release and increasing ocular bioavailability. The T_gel_ of the prepared formulations was assessed by rotational rheometry. The gelation temperature was evaluated by oscillation measurements performed at a constant frequency value, in a temperature range spanning the physiological temperature. All formulations displayed a stepwise increase in G′ and G″, from a liquid (G′ ≤ G″) to a gel state (G′ > G″), where both moduli reached a plateau (Figure S1). The formulation containing 22% *w*/*v* poloxamer 407 displayed a crossover of G′ and G″ at 24 °C (Figure S1a). However, the crossover could not be noticed for all samples, as demonstrated in Figure S1b for the formulation containing 17% *w*/*v* poloxamer 407. Therefore, the temperature at which the elastic modulus G′ attained the plateau value was considered as the T_gel_. Figure 2a shows the change in elastic modulus (G′) with the temperature for all the concentrations tested. The T_gel_ and G′ values remarkably depended on the polymer concentration (Figure 2b), ranging from 40 to 26 °C, and 300 to about 10,000 Pa, respectively, thus indicating that the formulation properties could be easily tuned to achieve viscoelastic properties more suitable to facilitate ocular application.

Compressive stress-strain measurements were carried out on the polymer solutions at 37 °C to evaluate the compressive strength and moduli, and the stability of the hydrogel formulations over repeated mechanical solicitations. The stress-strain curves (Figure 2c) showed a region of elastic deformation at low strain, followed by a plastic deformation zone, in which the stress did not change with increasing deformation values. Indeed, deformation caused chains to flow due to the absence of chemical crosslinks between the polymer segments. The compressive properties also depended strongly on the poloxamer 407 concentration, and the maximum stress values recorded varied from 0.5 (15% *w*/*v*) to 4 kPa (22% *w*/*v*). Similarly, the compressive modulus varied from 4.35 kPa (15% and 17% *w*/*v*), up to 23.5 and 52.5 kPa calculated for 19% and 22% *w*/*v*, respectively. As a comparison, the compressive modulus of the human cornea is about 1.7 MPa [42]. Cyclic compression tests indicated that the hydrogels had good mechanical stability upon repeated compression, as they maintained over 90% of the initial stress. Interestingly, all formulations showed a reproducible behavior upon unloading, attaining negative stress values, which demonstrate the cohesion of the solution and remarkable adhesive properties.

In the case of ocular administration, a shorter gelling time is preferred for faster onset of drug delivery. The gelling time depicts, in fact, the time required for the formation of the gel after administration, and this is fundamental for preventing its removal from the injection site, and prolonging the retention of the active substance in situ. The gelling time of all prepared batches was determined, by visual observation in simulated tear fluid at 34 °C. Gels with lower polymer concentration (15–17% *w*/*v*) had a longer gelation time of about 80 s, while gelation time was considerably shorter for those with higher polymer concentration (19–22% *w*/*v*) diminishing to 28.5 s. Figure 2e reports the visual observation of the gelling behavior of the poloxamer 407 at the optimized concentration of 19% *w*/*v*.

Based on rheological and gelling data, the RSV@Tgel formulation dispersing 1 mg RSV-NPs in 10 mL of 19% *w*/*v* poloxamer 407 (coded RSV@Tgel from here onward) was considered for further characterization. The physiological pH of the tears is approximately 7.4. Although eyes can tolerate a wide pH range (i.e., 4.5–8.5), the closer the pH of the formulation is to the physiological pH, the better it is tolerated. The pH values of all batches were around 6.8 and storage conditions, conducted at 4 and 25 °C for 14 days, had no significant influence on the corresponding pH of the preparation at different time points (Table 3). Moreover, the stability study of optimized in situ gel (RSV@Tgel) demonstrated that particle size and PDI were not influenced by storage temperature or time and were comparable with the initial values. In addition, the developed formulations remained clear throughout the stability storage period. On the contrary, RSV-NPs stored in free form showed a tendency to aggregate.

### 3.3. RSV Release from RSV@Tgel

**Figure 3:**
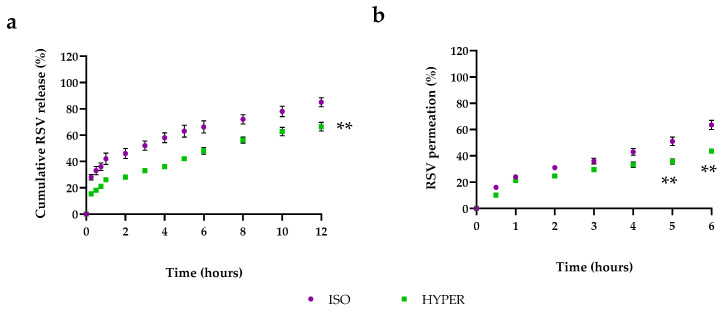
(**a**) Cumulative RSV release profile from RSV@tgel at 34 °C in isosmolar medium (ISO, 312 mOsM) or hyperosmolar medium (HYPER, 400 mOsM); Data were expressed as mean ± SD, *n* = 3. (Student’s *t*-test). ** *p* < 0.01 versus isosmolar medium at each point examined; (**b**) RSV permeation profile obtained from RSV@tgel in the above conditions. Data were expressed as mean ± SD, *n* = 3. (Student’s *t*-test). ** *p* < 0.01 versus isosmolar medium at 5 and 6 h time points.

Tear hyperosmolarity is one of the key pathogenesis mechanisms of DED. Therefore, to mimic in vivo dry eye conditions, the cumulative RSV release profiles were performed at 34 °C in an isosmolar medium (ISO, 312 mOsM) or hyperosmolar medium (HYPER, 400 mOsM). As depicted in Figure 3a, about 15% of the drug was released within 30 min by RSV@Tgel, reaching about 65% after 12 h in ISO condition. Furthermore, the RSV releasing rate from RSV-NPs was two-fold higher, with about 30% of RSV released after the same period, with a cumulative release percent almost achieving 85% at the end of the experiment. The low drug release from RSV@Tgel is probably due to the presence of a gel matrix formed at 35 °C that entraps the nanoparticles, slowing down drug diffusion from PLGA to the gel matrix, and subsequently through the gel into the medium [43]. In the case of nanoparticles alone, the small size led to the burst release of the majority of the encapsulated drugs over the first hours and slow release afterward. Despite the RSV-NPs high initial burst, it may be helpful to counteract inflammation in a short time, the following low RSV release requires repeated and close applications to maintain the therapeutic concentration. On the other hand, the sustained and long-term release phase of RSV@Tgel offers the possibility to steadily provide the bioactive response.

To obtain information about in vivo epithelium permeation, a Franz diffusion cell study was performed by using cellulose membrane simulating human cornea. The cumulative percentage of RSV in the receptor compartment was about 65% and 48% for RSV-NPs and RSV@Tgel, respectively, after 6 h (Figure 3b). The low corneal permeation of RSV@Tgel could be attributed to the presence of a hydrogel network that slowed down the permeation process. Similar results were obtained by Alruwaili et al. for gentamycin-loaded chitosan nanoparticles embedded into the carbopol hydrogel [44].

### 3.4. RSV@Tgel Protection of Corneal Cells from Oxidative Damage

The first prerequisite for a new ocular drug vehicle is to be innocuous or show quite low cellular toxicity. In this context, human corneal epithelial cells (HCECs) were incubated in the presence of different concentrations of RSV@Tgel conditioned medium (CM; 1:1, 1:2, and 1:5 *v*/*v*) for 24 h. As demonstrated in Figure S2a, the cell viability was around 98% for all tested concentrations. Moreover, less than 10% of lactate dehydrogenase (LDH) was detected in the culture medium (Figure S2b), confirming the negligible toxicity of the ocular gel to the cell membrane. As expected, Triton X-100, which was used as a positive control of membrane damage, dramatically decreased HCECs viability. Taken together, the results suggest an excellent safety profile of RSV@Tgel.

The ROS overproduction is induced on the ocular surface as a consequence of prolonged exposure to atmospheric oxygen and/or environmental factors (e.g., air pollution, wind, low humidity) leading to the loss of physiological balance between the production of free radicals and the ocular defense mechanisms [45,46,47]. Consequently, ROS accumulation can be responsible for damage to the ocular tissues. Recent studies have confirmed, both in vitro and in vivo, the involvement of oxidative stress in DED reporting the increase in ROS, lipid oxidative stress markers and inflammatory cells [48,49].

**Figure 4:**
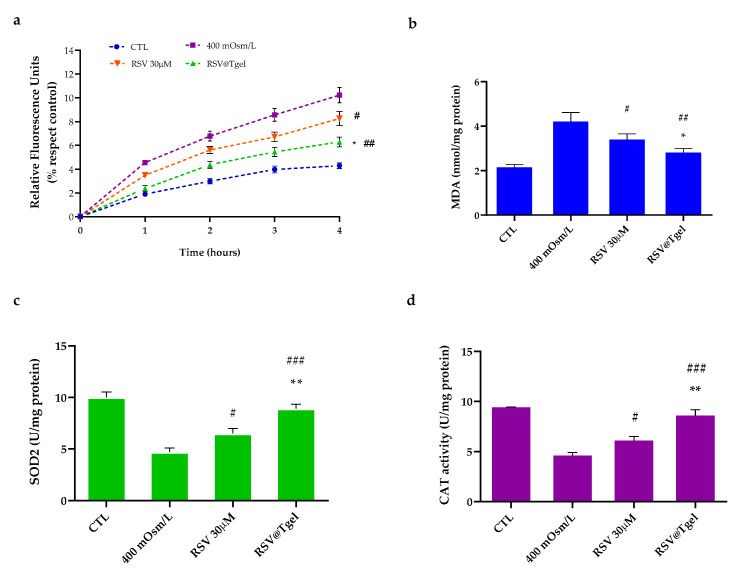
Effect of RSV@Tgel on ROS production. HCECs were cultured under hyperosmotic stress (400 mOsM) in presence of RSV (30 µM) or RSV@Tgel for 24 h. (**a**) Intracellular ROS release determined by oxidized H2DCFDA (DCF). (**b**) Quantification of Malondialdehyde used as a marker of lipid peroxidation. (**c**) Superoxide dismutase (SOD2) and (**d**) Catalase (CAT) activities measured by assay kit. Results are expressed as the means of three independent experiments ± S.D. (*n* = 3). Statistical analysis was performed by One-way ANOVA, * *p* < 0.05 and ** *p* < 0.01 versus RSV 30 μM; # *p* < 0.05, ## *p* < 0.01, and ### *p* < 0.001 versus 400 mOsM-treated cells.

To test whether RSV@Tgel was effective in reducing intracellular ROS production, an in vitro hyperosmolarity-induced dry eye model was used [46,50]. The hyperosmolarity of tear film has been recognized as a pro-inflammatory stress factor for the corneal epithelium, leading to cell apoptosis. According to a preliminary assessment, hyperosmolarity of 400 mOsm/L was identified as the correct balance between cell survival and death. The amount of intracellular ROS was defined based on the relative concentration of fluorescent 2′,7′-dichlorodihydrofluorescein (DCF). As shown in Figure 4a, hyperosmolar stress (400 mOsm/L) induces a substantial increase in HCE intracellular oxidants production approximately 2.4 times with respect to untreated cells (control). Pre-incubation with RSV alone (30 µM) induces a slight reduction in the DCF fluorescence intensity (around 20%, *p* < 0.05) with respect to the hyperosmotic group, whereas, in the presence of RSV@Tgel, ROS formation was markedly reduced (about 45%, *p* < 0.01) with a slight increase with respect to the normal isosmotic group.

Under oxidative stress, cellular membrane lipids are the prime targets of ROS attack. Lipid peroxidation leads to the formation of chemically reactive lipid aldehydes, such as MDA, capable of causing severe damage to nucleic acids and proteins, altering their functions and leading to the loss of both structural and metabolic function of cells [51]. As reported in Figure 4b, intracellular lipid peroxidation increased under hyperosmotic conditions by 1.9 times compared with the control. On the contrary, the amount of MDA diminished by 20% in cells treated with RSV alone, while a decrease of about 40% was noted in presence of RSV@Tgel.

Several clinical studies report that patients with ocular diseases have lower levels of SOD, catalase, and glutathione peroxidase (GPx) [52]. Indeed, SOD and CAT constitute, along with glutathione peroxidase, the first antioxidant defense system in cells acting as scavengers for O_2_^•−^ and H_2_O_2_. SOD converts superoxide to oxygen and hydrogen peroxide, and then CAT acts as a catalyst in the reaction of hydrogen peroxide decomposition to water and oxygen [53]. In normal eyes, antioxidant enzymes are highly expressed in the corneal and conjunctival epithelium, while in dry eye syndrome their expression is much less pronounced and is strongly related to the increasing severity of dry eye symptoms [54]. To further validate the role of released RSV in protecting corneal epithelial cells against oxidative damage triggered by hyperosmolarity, the activities of SOD and CAT were evaluated (Figure 4c,d). Hyperosmotic stress caused the reduction of both anti-oxidative enzymes (47% SOD, 52% CAT, respectively) compared to the control cells cultured in an isotonic environment, whereas the changes of activities were partially reversed by the pre-treatment with RSV@Tgel (32% SOD, 39% CAT, respectively) demonstrating a good ability to protect mitochondria from oxidative damage.

Pintea et al. have reported on the ability of resveratrol to prevent retinal pigment epithelium cell degeneration induced by oxidative stress [55]. Pretreatment with resveratrol at micromolar concentrations led to a dose-dependent increase in ROS-related enzyme activities (SOD, CAT and GHS), supporting the hypothesis that this polyphenol elicits antioxidant effects by directly scavenging the ROS in retinal pigment epithelium (RPE) cells. In another study, RSV significantly enhanced cell viability and promoted cell growth, by modulating superoxide dismutase (SOD)/malondialdehyde MDA) activity in RPE cells exposed to H_2_O_2_ [56].

### 3.5. Effect of RSV@Tgel on HCEC Mitochondrial Bioenergetics

Mitochondria are the major site of intracellular ROS production as well as the target of oxidative damage [57]. Since various research accounts report the involvement of mitochondrial dysfunction in DED progression [58], a real-time measurement of HCECs oxygen consumption rate (OCR) was performed using the seahorse XFp analyzer. In particular, key mitochondrial activities were evaluated, including basal respiration (detected as the baseline OCR before the addition of oligomycin), maximal respiration (evaluated as the OCR after the addition of FCCP), spare respiratory capacity (the difference between the maximal and basal respiration), and ATP turnover (the oligomycin-sensitive OCR).

**Figure 5:**
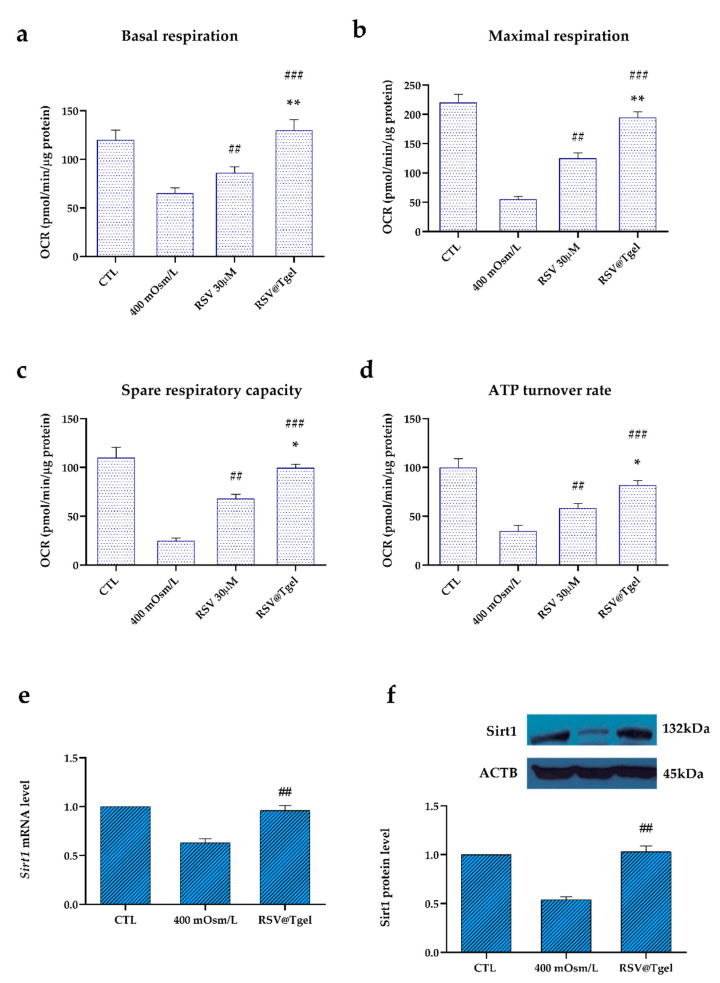
RSV@Tgel improves mitochondrial bioenergetics (**a**–**d**). HCECs were cultured in serum-free DMEM under hyperosmotic stress (400 mOsM) in presence of RSV (30 µM) or RSV@Tgel for 2 h. Measurements of OCR were taken in real-time using an XFe24 Flux Analyzer. OCRs were normalized to total protein content. Data are an average of three separate experiments. Statistical analysis was performed by One-way ANOVA, * *p* < 0.05 and ** *p* < 0.01 versus RSV 30 μM; ## *p* < 0.01, and ### *p* < 0.001 versus 400 mOsM-treated cells. Determination of Sirt1 gene expression (**e**) and protein (**f**) in HCECs under hyperosmotic stress or in presence of RSV@Tgel. Sirt1 mRNA levels were assessed by real-time PCR and calculated after normalization to *ACTB* mRNA levels. The protein levels of Sirt1 were quantified by Western blotting, and β-actin was used as the control protein. The protein levels were quantified using ImageJ. Statistical analysis was performed by the Student *t*-test ## *p* < 0.01 versus 400 mOsM-treated cells.

As expected, hyperosmotic conditions induced a reduction of about 50% (*p* < 0.01) in HCE cell mitochondrial bioenergetics with respect to untreated cells (Figure 5a–d). The presence of RSV alone is capable of partially protecting the mitochondrial function of HCE cells under hyperosmotic stress, with a recovery of about 30%. On the other hand, the treatment with RSV@Tgel led to a substantial increase of OCR in the measured respiration functions. In particular, in the case of ATP turnover rate, basal respiration, and maximal respiration, RSV@Tgel treatment restored the OCR at a level similar to that of untreated cells.

It has been demonstrated that RSV enhanced mitochondrial function by activating Sirt1, a highly conserved NAD^+^-dependent histone deacetylase [59] which regulates a wide range of cellular processes, including antioxidation, anti-apoptosis, DNA repair, antiaging, and life-span extension [60]. Recently, it has been found that abnormal Sirt1 expression provokes mitochondrial dysfunction, leading to ocular diseases, including cataracts, age-associated macular degeneration, diabetic retinopathy and glaucoma [61]. Under the hyperosmotic conditions, HCECs revealed a significant downregulation of Sirt1 at both mRNA and protein levels, of about 38% and 49%, respectively, compared to untreated cells (Figure 5e,f). The presence of RSV@Tgel restores the expression of Sirt1 (mRNA and protein) at levels not significantly different from untreated cells. Our results are in line with the literature reporting that RSV promotes mitochondrial protection against oxidation-induced toxicity through the increase in mitochondrial bioenergetics [62,63]. Moreover, Chen et al. demonstrated that high osmotic-induced mitochondrial dysfunction was reduced after treatment with RSV 50 µM by inducing Sirt1 upregulation [64].

### 3.6. RSV@Tgel Suppression of Inflammatory Response under Hyperosmotic Stress

Recent studies in both animal- and cell-based DED models identify hyperosmotic stress as a principal pathogenic factor responsible for the production of inflammatory signaling molecules, including interleukin, tumor necrosis factor, and matrix metalloproteinases [65,66]. The induced-inflammatory state gives rise to further cell damage, such as apoptosis of conjunctival, corneal, and lacrimal gland epithelial cells, initiating a cycle of events that perpetuate the DED condition. In this context, molecules able to interrupt the vicious circle of DED pathologies, such as polyphenols, and RSV in particular, could be relevant.

**Figure 6:**
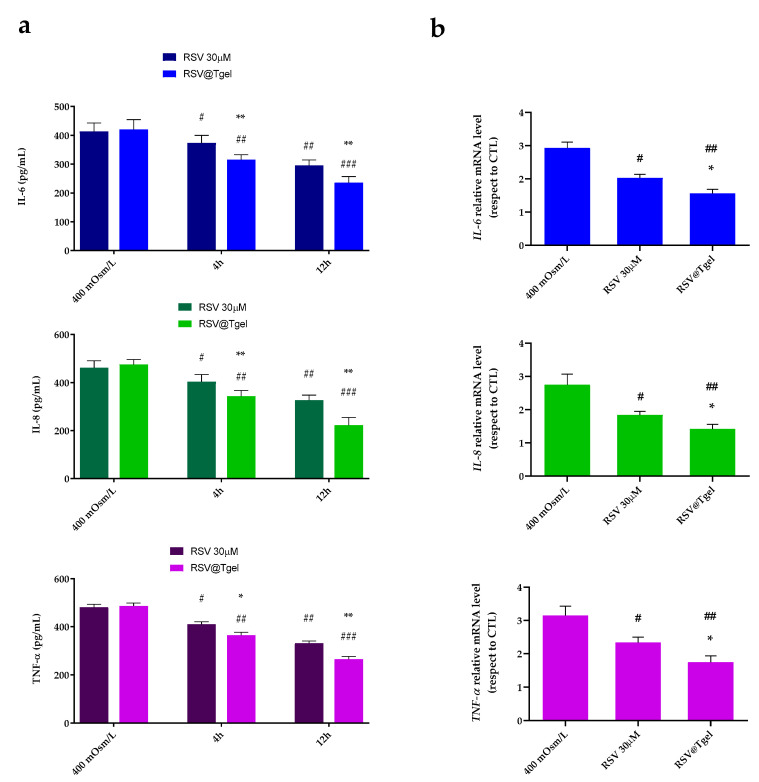
RSV suppressed expression of pro-inflammatory cytokines IL-1 β, IL-6, and TNF-α in HCECs exposed to hyperosmotic medium. HCECs were cultured in isomolar (292 mOsm/L) medium, then switched to hyperosmotic medium (400 mOsm/L) alone or in the presence of RSV and RSV@Tgel for 24 h to measure protein level by ELISA (**a**); or for 4 h to evaluate mRNA level by RT-qPCR (**b**). Data were summarized as mean ± SD from three separate experiments. * *p* < 0.05, ** *p* < 0.01 vs. cells exposed to 400 mOsM/L; # *p* < 0.05, ## *p* < 0.01, and ### *p* < 0.001 vs. RSV@Tgel (Student’s *t*-test).

As shown in Figure 6a, exposure of HCECs to the hyperosmotic medium resulted in a significant increase in IL-1β, IL-6, and TNF-α production (three-fold, *p* < 0.001). Pre-incubation of cells with 30 μM RSV and RSV@Tgel significantly reduced the amount of released cytokines under hyperosmotic stress by about 30% and 41% for IL-6; about 20% and 50% for IL-8 and TNF-α, respectively. In response to stress stimulation, the mRNA levels of all tested cytokines (relative to the housekeeping gene) were significantly upregulated (two-fold *p* < 0.01) in damage caused by treatment with cells, compared with the control group (Figure 6b). Consistently, the protective effects of RSV result in a reduction in the expression levels of all interleukins tested, by around 30% for RSV alone, which reaches 65% in the presence of RSV@Tgel.

These results suggest that RSV has a suppressive effect on inflammatory biomarkers at both mRNA and protein levels in ocular surface epithelial cells. Our results are in agreement with several studies that demonstrate RSV involvement, both in vitro and in animal experimentation, in reducing pro-inflammatory mediators and cellular pathways involved in inflammation [67,68]. Hsu and colleagues demonstrated the anti-inflammatory effects of RSV on a myopia animal model with a reduction of the expression levels of matrix metalloproteinase 2 (MMP2), transforming growth factor (TGF)-β, and nuclear factor (NF)-κB [69]. Moreover, the treatment with RSV induced the downregulation of inflammatory cytokine production, and the inhibition of AKT, c-Raf, Stat3, and NFκB phosphorylation in ARPE19 cells. Luna et al. investigated the therapeutic effects of RSV administration in chronic oxidative stress-induced trabecular meshwork cells [70]. Their results demonstrated that resveratrol effectively decreased the production of intracellular ROS (iROS) and inflammatory markers such as interleukin-1 alpha (IL-1α), interleukin (IL)-6, and IL-8. Moreover, RSV, via vitamin D receptors, was able to reduce oxidative stress in human corneal epithelial cells under hyperosmolar conditions through the activation of Notch signaling.

## 4. Conclusions

DED is a dynamic and complex disease that can cause significant damage to the ocular surface. Several studies demonstrated the potential applicability of RSV as a treatment for DED. However, the low RSV water solubility and stability, and its poor ocular bioavailability limit its clinical application. This study provides new insights into the therapeutic effects of intra-ocular instillation of thermosensitive hydrogels (RSV@Tgel) loaded with acetylated PLGA-PEI nanoparticles able to allow the sustained delivery of resveratrol (RSV-NPs). RSV released from the hydrogel protects HCECs from ROS damage and reverts the activation of inflammatory factors, limiting the damage caused by hyperosmotic conditions. Moreover, RSV@Tgel protects mitochondria from oxidation-induced toxicity through the increase in mitochondrial bioenergetics and Sirt1 upregulation. Overall, the reported results indicate that RSV@Tgel acts as an adjuvant in dry eye disease because of its effect against inflammation and oxidative stress. Furthermore, the thermosensitive formulation extends the residence time in the eye, thus allowing to reduce the number of eye drop instillations, and increasing patient compliance.

## Figures and Tables

**Table 1:** Primers used for qRT-PCR.

Gene	Accession Number	Forward (5′-3′)	Reverse (5′-3′)
*IL-6*	NM_000600.5	CGCCTTCGGTCCAGTTGCC	GCCAGTGCCTCTTTGCTGCTTT
*IL-8*	NM_000584.4	CTCTTGGCAGCCTTCCTGATTTC	TTTTCCTTGGGGTCCAGACAGAG
*TNF-α*	NM_000594.4	AACATCCAACCTTCCCAAACGC	TGGTCTCCAGATTCCAGATGTCAGG
*Sirt1*	NM_012238.2	GCCTCACATGCAAGCTCTAGTGAC	TTCGAGGATCTGTGCCAATCATAA
*ACTB*	NM_001101.5	ACTCTTCCAGCCTTCCTTCC	CGTACAGGTCTTTGCGGATG

**Table 2:** Particle size, zeta potential, polydispersity index, as measured by DLS, and encapsulation efficiency of nanoparticles.

Sample	Polymer (mg)	RSV (mg)	Z-Average (nm)	Zeta Potential (mV)	Polydispersity Index (PDI)	Encapsulation Efficiency (EE %)
NPs	100	0	96.3 ± 0.8	21.9 ± 1.6	0.21 ± 0.6	
RSV_1_-NPs	100	5	89.2 ± 0.9	20.7 ± 1.9	0.19 ± 0.9	46.8 ± 2.8
RSV_2_-NPs	100	10	125.4 ± 1.3	22.1 ± 1.3	0.16 ± 0.3	78.3 ± 4.9
RSV_3_-NPs	100	20	148.7 ± 2.2	21.6 ± 1.8	0.22 ± 1.2	66.4 ± 5.9

**Table 3:** Stability studies of RSV-NPs and RSV@Tgel before and after 14-day storage.

	RSV-NPs (0 Day)		RSV-NPs (14 Days)		RSV@Tgel (0 Day)		RSV@Tgel (14 Days)	
	4 °C	25 °C	4 °C	25 °C	4 °C	25 °C	4 °C	25 °C
Average particle size (nm ± S.D.)	126.7 ± 9.1	129.6 ± 3.1	169.18 ± 19.2	176.6 ± 11.4	135.4 ± 9.0	138.7 ± 6.4	137.6 ± 5.3	139.1 ± 4.3
PDI	0.15 ± 0.0	0.17 ± 0.0	0.4 ± 0.0	0.5 ± 0.0	0.11 ± 0.0	0.14 ± 0.0	0.13 ± 0.0	0.12 ± 0.0
pH	6.7 ± 0.2	6.6 ± 0.2	6.6 ± 0.1	6.7 ± 0.1	6.8 ± 0.2	6.7 ± 0.3	6.7 ± 0.2	6.7 ± 0.6

Note: Data were expressed as mean ± standard deviation, *n* = 3.

## Data Availability

The data presented in this study are available in the article.

## Supplementary Materials

The following supporting information can be downloaded at: https://www.mdpi.com/article/10.3390/antiox12050993/s1, Figure S1: Evolution of the dynamic storage and dissipative moduli (G′ and G″) in non-isothermal oscillatory shear experiments of RSV@Tgel formulations containing (a) 22%, and (b) 17% *w*/*v* poloxamer 407. Figure S2: In vitro biocompatibility of RSV@Tgel after 24, 48, and 72 h. Cell viability (a) and Lactate Dehydrogenase (LDH) release (b) were assessed in HCECs incubated with different concentrations of RSV@Tgel conditioned medium (CM; 1:1, 1:2, and 1:5 *v*/*v*). Untreated cells and Triton X-100 treated cells were considered as a negative and positive control, respectively. Results are expressed as the mean of three independent experiments ± S.D. (*n* = 3).

## References

[1] Berg E.J., Ying G.S., Maguire M.G., Sheffield P.E., Szczotka-Flynn L.B., Asbell P.A., Shen J.F., Group D.S.R. (2020). Climatic and Environmental Correlates of Dry Eye Disease Severity: A Report From the Dry Eye Assessment and Management (DREAM) Study. Transl. Vis. Sci. Technol..

[2] Dogru M., Kojima T., Simsek C., Tsubota K. (2018). Potential Role of Oxidative Stress in Ocular Surface Inflammation and Dry Eye Disease. Investig. Ophthalmol. Vis. Sci..

[3] Yamaguchi T. (2018). Inflammatory Response in Dry Eye. Investig. Ophthalmol. Vis. Sci..

[4] Shimazaki J. (2018). Definition and Diagnostic Criteria of Dry Eye Disease: Historical Overview and Future Directions. Investig. Ophthalmol. Vis. Sci..

[5] Gurnani B., Kaur K. (2021). Current approach in surgical management of dry eyes—Dry eye review II. TNOA J. Ophthalmic Sci. Res..

[6] Mohamed H.B., Abd El-Hamid B.N., Fathalla D., Fouad E.A. (2022). Current trends in pharmaceutical treatment of dry eye disease: A review. Eur. J. Pharm.Sci. Off. J. Eur. Fed. Pharm. Sci..

[7] De Luca I., Di Cristo F., Valentino A., Peluso G., Di Salle A., Calarco A. (2022). Food-Derived Bioactive Molecules from Mediterranean Diet: Nanotechnological Approaches and Waste Valorization as Strategies to Improve Human Wellness. Polymers.

[8] Xu Z., Sun T., Li W., Sun X. (2017). Inhibiting effects of dietary polyphenols on chronic eye diseases. J. Funct. Foods.

[9] Bungau S., Abdel-Daim M.M., Tit D.M., Ghanem E., Sato S., Maruyama-Inoue M., Yamane S., Kadonosono K. (2019). Health Benefits of Polyphenols and Carotenoids in Age-Related Eye Diseases. Oxidative Med. Cell. Longev..

[10] Choi W., Lee J.B., Cui L., Li Y., Li Z., Choi J.S., Lee H.S., Yoon K.C. (2016). Therapeutic Efficacy of Topically Applied Antioxidant Medicinal Plant Extracts in a Mouse Model of Experimental Dry Eye. Oxidative Med. Cell. Longev..

[11] Favero G., Moretti E., Krajčíková K., Tomečková V., Rezzani R. (2021). Evidence of Polyphenols Efficacy against Dry Eye Disease. Antioxidants.

[12] Abu-Amero K.K., Kondkar A.A., Chalam K.V. (2016). Resveratrol and Ophthalmic Diseases. Nutrients.

[13] Bryl A., Falkowski M., Zorena K. (2022). The Role of Resveratrol in Eye Diseases-A Review of the Literature. Nutrients.

[14] Gao Y., Fu R., Wang J., Yang X., Wen L., Feng J. (2018). Resveratrol mitigates the oxidative stress mediated by hypoxic-ischemic brain injury in neonatal rats via Nrf2/HO-1 pathway. Pharm. Biol..

[15] Vivero-Lopez M., Sparacino C., Quelle-Regaldie A., Sánchez L., Candal E., Barreiro-Iglesias A., Huete-Toral F., Carracedo G., Otero A., Concheiro A. (2022). Pluronic^®^/casein micelles for ophthalmic delivery of resveratrol: In vitro, ex vivo, and in vivo tests. Int. J. Pharm..

[16] Castro B.F.M., Fulgêncio G.D.O., Domingos L.C., Cotta O.A.L., Silva-Cunha A., Fialho S.L. (2020). Positively charged polymeric nanoparticles improve ocular penetration of tacrolimus after topical administration. J. Drug Deliv. Sci. Technol..

[17] Conte R., De Luise A., Valentino A., Di Cristo F., Petillo O., Riccitiello F., Di Salle A., Calarco A., Peluso G. (2018). Hydrogel Nanocomposite Systems: Characterization and Application in Drug-Delivery Systems. Nanocarriers for Drug Delivery: Nanoscience and Nanotechnology in Drug Delivery.

[18] Hamcerencu M., Desbrieres J., Popa M., Riess G. (2020). Thermo-sensitive gellan maleate/N-isopropylacrylamide hydrogels: Initial “in vitro” and “in vivo” evaluation as ocular inserts. Polym. Bull..

[19] Conte R., Finicelli M., Borrone A., Margarucci S., Peluso G., Calarco A., Bosetti M. (2023). MMP-2 Silencing through siRNA Loaded Positively-Charged Nanoparticles (AcPEI-NPs) Counteracts Chondrocyte De-Differentiation. Polymers.

[20] Valentino A., Conte R., De Luca I., Di Cristo F., Peluso G., Bosetti M., Calarco A. (2022). Thermo-Responsive Gel Containing Hydroxytyrosol-Chitosan Nanoparticles (Hyt@tgel) Counteracts the Increase of Osteoarthritis Biomarkers in Human Chondrocytes. Antioxidants.

[21] Dyawanapelly S., Koli U., Dharamdasani V., Jain R., Dandekar P. (2016). Improved mucoadhesion and cell uptake of chitosan and chitosan oligosaccharide surface-modified polymer nanoparticles for mucosal delivery of proteins. Drug Deliv. Transl. Res..

[22] Matthew J.E., Nazario Y.L., Roberts S.C., Bhatia S.R. (2002). Effect of mammalian cell culture medium on the gelation properties of Pluronic F127. Biomaterials.

[23] Khattab A., Marzok S., Ibrahim M. (2019). Development of optimized mucoadhesive thermosensitive pluronic based in situ gel for controlled delivery of Latanoprost: Antiglaucoma efficacy and stability approaches. J. Drug Deliv. Sci. Technol..

[24] Amaghnouje A., Mechchate H., Es-Safi I., Boukhira S., Aliqahtani S.A., MNoman O., ANasr F., Conte R., Calarco A., Bousta D. (2020). Subacute Assessment of the Toxicity and Antidepressant-Like Effects of *Origanum majorana* L. Polyphenols in Swiss Albino Mice. Molecules.

[25] Bao Q., Newman B., Wang Y., Choi S., Burgess D.J. (2018). In vitro and ex vivo correlation of drug release from ophthalmic ointments. J. Control. Release.

[26] Shetty R., Subramani M., Murugeswari P., Anandula V.R., Matalia H., Jayadev C., Ghosh A., Das D. (2020). Resveratrol Rescues Human Corneal Epithelial Cells Cultured in Hyperosmolar Conditions: Potential for Dry Eye Disease Treatment. Cornea.

[27] Di Cristo F., Valentino A., De Luca I., Peluso G., Bonadies I., Calarco A., Di Salle A. (2022). PLA Nanofibers for Microenvironmental-Responsive Quercetin Release in Local Periodontal Treatment. Molecules.

[28] Conte R., De Luca I., Valentino A., Cerruti P., Pedram P., Cabrera-Barjas G., Moeini A., Calarco A. (2023). Hyaluronic Acid Hydrogel Containing Resveratrol-Loaded Chitosan Nanoparticles as an Adjuvant in Atopic Dermatitis Treatment. J. Funct. Biomater..

[29] De Luca I., Di Salle A., Alessio N., Margarucci S., Simeone M., Galderisi U., Calarco A., Peluso G. (2016). Positively charged polymers modulate the fate of human mesenchymal stromal cells via ephrinB2/EphB4 signaling. Stem Cell Res..

[30] Di Cristo F., Calarco A., Digilio F.A., Sinicropi M.S., Rosano C., Galderisi U., Melone M.A.B., Saturnino C., Peluso G. (2020). The Discovery of Highly Potent THP Derivatives as OCTN2 Inhibitors: From Structure-Based Virtual Screening to In Vivo Biological Activity. Int. J. Mol. Sci..

[31] Melone M.A.B., Calarco A., Petillo O., Margarucci S., Colucci-D’Amato L., Galderisi U., Koverech G., Peluso G. (2013). Mutant huntingtin regulates EGF receptor fate in non-neuronal cells lacking wild-type protein. Biochim. Biophys. Acta (BBA)—Mol. Basis Dis..

[32] Di Cristo F., Finicelli M., Digilio F.A., Paladino S., Valentino A., Scialò F., D’Apolito M., Saturnino C., Galderisi U. (2019). Meldonium improves Huntington’s disease mitochondrial dysfunction by restoring peroxisome proliferator-activated receptor γ coactivator 1α expression. J. Cell. Physiol..

[33] Gorantla S., Rapalli V.K., Waghule T., Singh P.P., Dubey S.K., Saha R.N., Singhvi G. (2020). Nanocarriers for ocular drug delivery: Current status and translational opportunity. RSC Adv..

[34] Mohsen A.M. (2022). Cationic Polymeric Nanoparticles for Improved Ocular Delivery and Antimycotic Activity of Terconazole. J. Pharm. Sci..

[35] Rivas C., Tarhini M., Badri W., Miladi K., Greige-Gerges H., Nazari Q.A., Galindo S., Alvarez Roman R., Fessi H., Elaissari A. (2017). Nanoprecipitation process: From encapsulation to drug delivery. Int. J. Pharm..

[36] Chen Z., Tai Z., Gu F., Hu C., Zhu Q., Gao S. (2016). Aptamer-mediated delivery of docetaxel to prostate cancer through polymeric nanoparticles for enhancement of antitumor efficacy. Eur. J. Pharm. Biopharm..

[37] Chhonker Y.S., Prasad Y.D., Chandasana H., Vishvkarma A., Mitra K., Shukla P.K., Bhatta R.S. (2015). Amphotericin-B entrapped lecithin/chitosan nanoparticles for prolonged ocular application. Int. J. Biol. Macromol..

[38] Georgiev G.A., Eftimov P., Yokoi N. (2019). Contribution of Mucins towards the Physical Properties of the Tear Film: A Modern Update. Int. J. Mol. Sci..

[39] Holland E.J., Darvish M., Nichols K.K., Jones L., Karpecki P.M. (2019). Efficacy of topical ophthalmic drugs in the treatment of dry eye disease: A systematic literature review. Ocul. Surf..

[40] Pereira G.G., Dimer F.A., Guterres S.S., Kechinski C.P., Granada J.E., Cardozo N.S.M. (2013). Formulation and characterization of poloxamer 407^®^: Thermoreversible gel containing polymeric microparticles and hyaluronic acid. Química Nova.

[41] Carlfors J., Edsman K., Petersson R., Jörnving K. (1998). Rheological evaluation of Gelrite in situ gels for ophthalmic use. Eur. J. Pharm. Sci. Off. J. Eur. Fed. Pharm. Sci..

[42] Sharifi S., Islam M.M., Sharifi H., Islam R., Koza D., Reyes-Ortega F., Alba-Molina D., Nilsson P.H., Dohlman C.H., Mollnes T.E. (2021). Tuning gelatin-based hydrogel towards bioadhesive ocular tissue engineering applications. Bioact. Mater..

[43] Rupenthal I.D., Green C.R., Alany R.G. (2011). Comparison of ion-activated in situ gelling systems for ocular drug delivery. Part 1: Physicochemical characterisation and in vitro release. Int. J. Pharm..

[44] Alruwaili N.K., Zafar A., Imam S.S. (2020). Stimulus Responsive Ocular Gentamycin-Ferrying Chitosan Nanoparticles Hydrogel: Formulation Optimization, Ocular Safety and Antibacterial Assessment. Int. J. Nanomed..

[45] Mandell J.T., Idarraga M., Kumar N., Galor A. (2020). Impact of Air Pollution and Weather on Dry Eye. J. Clin. Med..

[46] Park B., Jo K., Lee T.G. (2019). Polydatin Inhibits NLRP3 Inflammasome in Dry Eye Disease by Attenuating Oxidative Stress and Inhibiting the NF-κB Pathway. Nutrients.

[47] Deng R., Hua X., Li J., Chi W., Zhang Z., Lu F., Zhang L., Pflugfelder S.C., Li D.Q. (2015). Oxidative stress markers induced by hyperosmolarity in primary human corneal epithelial cells. PLoS ONE.

[48] Wakamatsu T.H., Dogru M., Ayako I., Takano Y., Matsumoto Y., Ibrahim O.M., Okada N., Satake Y., Fukagawa K., Shimazaki J. (2010). Evaluation of lipid oxidative stress status and inflammation in atopic ocular surface disease. Mol. Vis..

[49] Zheng Q., Ren Y., Reinach P.S., She Y., Xiao B., Hua S., Qu J., Chen W. (2014). Reactive oxygen species activated NLRP3 inflammasomes prime environment-induced murine dry eye. Exp. Eye Res..

[50] Wang H.H., Chen W.Y., Huang Y.H., Hsu S.M., Tsao Y.P., Hsu Y.H., Chang M.S. (2022). Interleukin-20 is involved in dry eye disease and is a potential therapeutic target. J. Biomed. Sci..

[51] Shi Q., Vaillancourt F., Côté V., Fahmi H., Lavigne P., Afif H., Di Battista J.A., Fernandes J.C., Benderdour M. (2006). Alterations of metabolic activity in human osteoarthritic osteoblasts by lipid peroxidation end product 4-hydroxynonenal. Arthritis Res. Ther..

[52] Cabrera M.P., Chihuailaf R.H. (2011). Antioxidants and the integrity of ocular tissues. Vet. Med. Int..

[53] Bhuyan K.C., Bhuyan D.K. (1970). Catalase in Ocular Tissue and Its Intracellular Distribution in Corneal Epithelium. Am. J. Ophthalmol..

[54] Cejková J., Ardan T., Simonová Z., Cejka C., Malec J., Dotrelová D., Brunová B. (2008). Decreased expression of antioxidant enzymes in the conjunctival epithelium of dry eye (Sjögren’s syndrome) and its possible contribution to the development of ocular surface oxidative injuries. Histol. Histopathol..

[55] Pintea A., Rugină D., Pop R., Bunea A., Socaciu C., Diehl H.A. (2011). Antioxidant effect of trans-resveratrol in cultured human retinal pigment epithelial cells. J. Ocul. Pharmacol. Ther. Off. J. Assoc. Ocul. Pharmacol. Ther..

[56] Yang Y., Wu Z.Z., Cheng Y.L., Lin W., Qu C. (2019). Resveratrol protects against oxidative damage of retinal pigment epithelium cells by modulating SOD/MDA activity and activating Bcl-2 expression. Eur. Rev. Med. Pharmacol. Sci..

[57] Uchino Y., Kawakita T., Miyazawa M., Ishii T., Onouchi H., Yasuda K., Ogawa Y., Shimmura S., Ishii N., Tsubota K. (2012). Oxidative stress induced inflammation initiates functional decline of tear production. PLoS ONE.

[58] Seen S., Tong L. (2018). Dry eye disease and oxidative stress. Acta Ophthalmol..

[59] Chen Y., Zhang H., Ji S., Jia P., Chen Y., Li Y., Wang T. (2021). Resveratrol and its derivative pterostilbene attenuate oxidative stress-induced intestinal injury by improving mitochondrial redox homeostasis and function via SIRT1 signaling. Free Radic. Biol. Med..

[60] Mimura T., Kaji Y., Noma H., Funatsu H., Okamoto S. (2013). The role of SIRT1 in ocular aging. Exp. Eye Res..

[61] Zhou M., Luo J., Zhang H. (2018). Role of Sirtuin 1 in the pathogenesis of ocular disease (Review). Int. J. Mol. Med..

[62] Sheu S.J., Liu N.C., Ou C.C., Bee Y.S., Chen S.C., Lin H.C., Chan J.Y. (2013). Resveratrol stimulates mitochondrial bioenergetics to protect retinal pigment epithelial cells from oxidative damage. Investig. Ophthalmol. Vis. Sci..

[63] Averilla J.N., Oh J., Kim J.-S. (2019). Carbon Monoxide Partially Mediates Protective Effect of Resveratrol Against UVB-Induced Oxidative Stress in Human Keratinocytes. Antioxidants.

[64] Chen J., Zhang W., Zheng Y., Xu Y. (2022). Ameliorative Potential of Resveratrol in Dry Eye Disease by Restoring Mitochondrial Function. Evid.-Based Complement. Altern. Med..

[65] Corrales R.M., Luo L., Chang E.Y., Pflugfelder S.C. (2008). Effects of osmoprotectants on hyperosmolar stress in cultured human corneal epithelial cells. Cornea.

[66] Baudouin C., Aragona P., Messmer E.M., Tomlinson A., Calonge M., Boboridis K.G., Akova Y.A., Geerling G., Labetoulle M., Rolando M. (2013). Role of hyperosmolarity in the pathogenesis and management of dry eye disease: Proceedings of the OCEAN group meeting. Ocul. Surf..

[67] Minagawa T., Okui T., Takahashi N., Nakajima T., Tabeta K., Murakami S., Yamazaki K. (2015). Resveratrol suppresses the inflammatory responses of human gingival epithelial cells in a SIRT1 independent manner. J. Periodontal Res..

[68] Goutham G., Manikandan R., Beulaja M., Thiagarajan R., Arulvasu C., Arumugam M., Setzer W.N., Daglia M., Nabavi S.F., Nabavi S.M. (2017). A focus on resveratrol and ocular problems, especially cataract: From chemistry to medical uses and clinical relevance. Biomed. Pharmacother..

[69] Hsu Y.A., Chen C.S., Wang Y.C., Lin E.S., Chang C.Y., Chen J.J., Wu M.Y., Lin H.J., Wan L. (2021). Anti-Inflammatory Effects of Resveratrol on Human Retinal Pigment Cells and a Myopia Animal Model. Curr. Issues Mol. Biol..

[70] Luna C., Li G., Liton P.B., Qiu J., Epstein D.L., Challa P., Gonzalez P. (2009). Resveratrol prevents the expression of glaucoma markers induced by chronic oxidative stress in trabecular meshwork cells. Food Chem. Toxicol..

