# Supplementary material for: In-Situ Thermoresponsive Hydrogel Containing Resveratrol-Loaded Nanoparticles as a Localized Drug Delivery Platform for Dry Eye Disease
Source: Antioxidants (Basel). 2023 Apr 25;12(5):993. doi: 10.3390/antiox12050993 (PMC10215589; doi:10.3390/antiox12050993)
Supplement: Supplementary file 1 [file antioxidants-12-00993-s001.zip › antioxidants-2333412-supplementary.pdf]

Figure S1

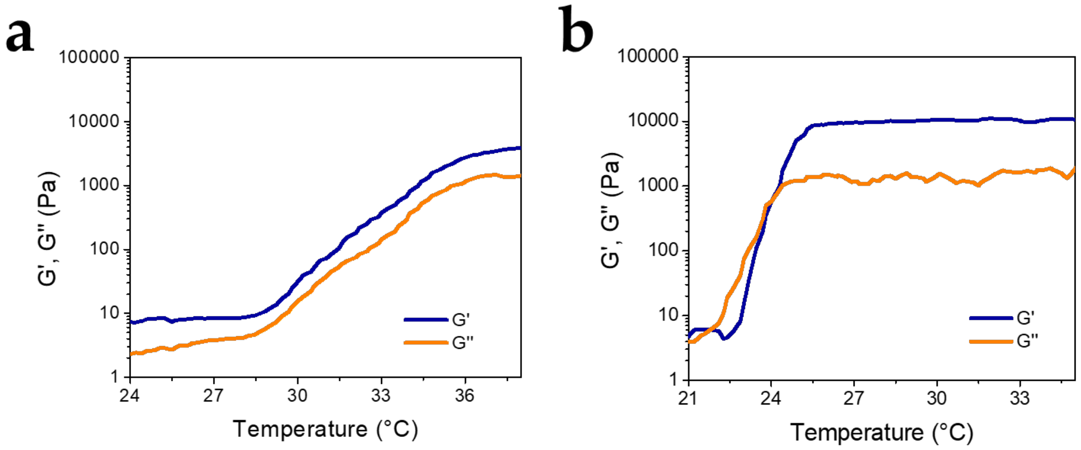

Figure S1. Evolution of the dynamic storage and dissipative moduli (G′ and G′′) in non-isothermal oscillatory shear experiments of RSV@Tgel formulations containing (a) 22%, and (b) 17% w/v poloxamer 407.

Figure S2

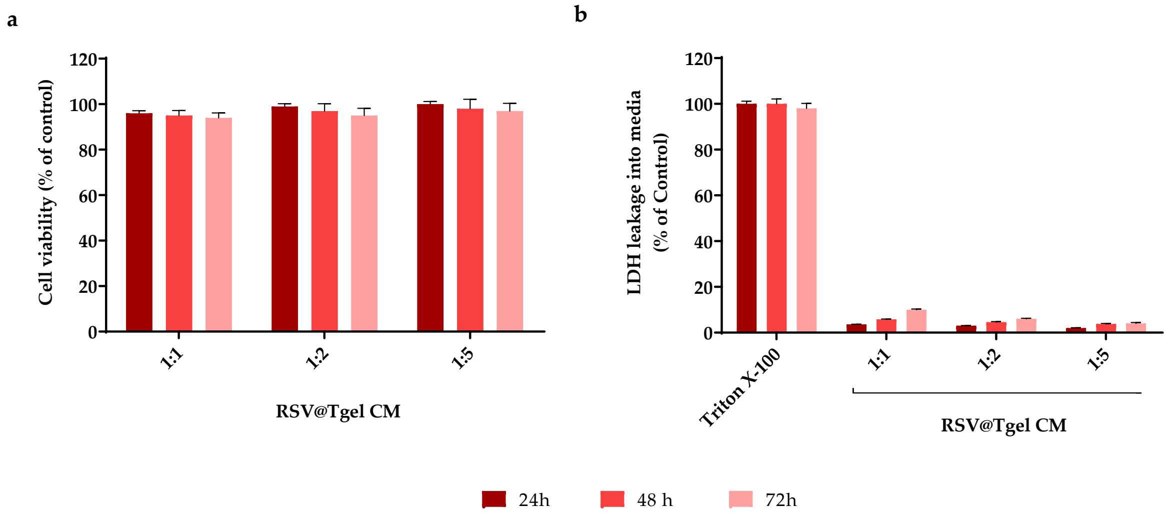

Figure S2. In vitro biocompatibility of RSV@Tgel after 24, 48, and 72 h. Cell viability (A) and Lactate Dehydrogenase (LDH) release (B) were assessed in HCECs incubated with different concentrations of RSV@Tgel conditioned medium (CM; 1:1, 1:2, and 1:5 v/v). Untreated cells and Triton X-100 treated cells were considered as negative and positive control, respectively. Results are expressed as the mean of three independent experiments ± S.D (n = 3)
